# Single-center experience of induction therapy in non-systemic vasculitic neuropathy

**DOI:** 10.1186/s42466-022-00198-5

**Published:** 2022-08-15

**Authors:** Christian Schneider, Meike K. Wassermann, Gereon R. Fink, Helmar C. Lehmann

**Affiliations:** 1 Department of Neurology, Medical Faculty and University Hospital of Cologne, Kerpener Straße 62, D-50937 Cologne, Germany; 2 Research Center Jülich, Institute of Neuroscience and Medicine (INM-3), Jülich, Germany

**Keywords:** Vasculitic neuropathy, Polyneuropathy, Multifocal neuropathy, Cyclophosphamide

## Abstract

**Background:**

No controlled studies for non-systemic vasculitic neuropathy treatment exist (NSVN). We compared the treatment response to induction therapy commonly used in clinical practice in NSVN.

**Methods:**

In this retrospective single-center study, 43 patients with biopsy-proven NSVN were analyzed. Patients were subdivided into groups depending on their initial treatment. Relapse rates, changes of motor and sensory symptoms, adverse events, predictors of relapses, and second-line treatment were compared.

**Results:**

Initial treatment regimens were corticosteroid monotherapy, cyclophosphamide monotherapy, pulsed corticosteroid therapy, and combination therapy. Discontinuation due to adverse events occurred in 6 of 43 patients. Clinical data did not differ between treatment groups. Within 12 months, 24.3% of patients relapsed. The median time to relapse was 4 (1.5, 6) months. No relapse occurred in the combination therapy group. However, there was no statistically significant difference in relapse-free survival between treatment groups (*p* = 0.58). Neither clinical data nor biopsy analysis predicted relapses sufficiently. As a second-line treatment, cyclophosphamide as mono- or combination therapy was used (7 of 9 patients) most frequently. One patient was treated with methotrexate, and one with IVIG.

**Conclusions:**

Induction therapy used in clinical practice is effective and mainly well-tolerated in NSVN. Our data do not support an overall advantage of cyclophosphamide over corticosteroid monotherapy. Controlled trials comparing the effectiveness of induction and maintenance therapy in NSVN are warranted.

**Supplementary Information:**

The online version contains supplementary material available at 10.1186/s42466-022-00198-5.

## Background

Non-systemic vasculitic neuropathy (NSVN) is a frequent cause of a chronic progressive neuropathy with a multifocal distribution of symptoms and electrophysiological characteristics of a predominantly axonal nerve damage pattern. Pain is frequently reported, and attack-like deterioration may occur [2]. Biopsy findings reveal an autoimmune-mediated inflammation of nerve adjacent vessels [6, 12]. Therapeutic strategies are mainly based on clinical experience, as no controlled trials exist, and retrospective cohort studies [1, 4, 13]. Although clinical experience and recent guidelines suggest that all NSVN patients should receive immunosuppressive therapy, precise treatment strategies are still a subject of debate [2]. In NSVN, induction therapy is recommended with corticosteroids only or, in severe cases, combined with cyclophosphamide (CYC), methotrexate (MTX), or azathioprine. In severe cases, pulsed intravenous corticosteroids or rituximab are discussed [1]. However, limited evidence exists about which approach has the most favorable balance of benefits and risks. Especially alternative treatment considerations derived from clinical experience, e.g., CYC-monotherapy, have not been investigated. These approaches are of particular interest as they might reduce infectious adverse events compared to combination therapy [3].

In our retrospective, single-center observational study, we analyzed data on induction therapy of 43 patients diagnosed with NSVN from 2015 to 2020.

## Methods

### Patient selection

**Fig. 1:**
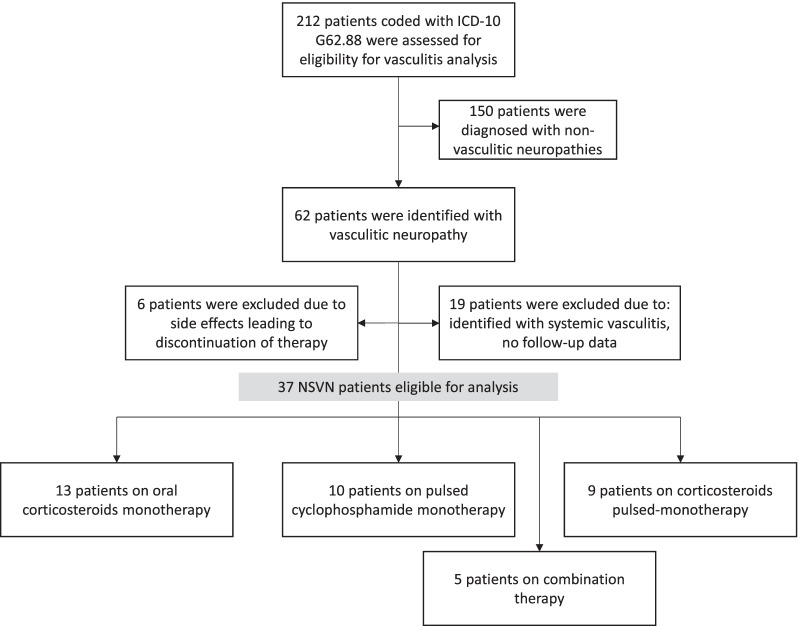
Inclusion process for analysis of induction therapy in patients with non-systemic vasculitic neuropathy (NSVN)

This retrospective analysis of induction therapy in patients with NSVN was conducted at the Department of Neurology at the University Hospital of Cologne. Patients diagnosed with polyneuropathy assigned to coding ICD-10 G62.88 (i.e., “other specified polyneuropathies”) of the International Statistical Classification of Diseases and Related Health Problems as the principal diagnosis from 2015 to 2020 were screened for eligibility. Clinical data, including neurophysiological examinations and neuropathological results of nerve biopsies, were retrieved from medical reports. Eligible patients had been diagnosed with a pathologically confirmed NSVN (pathologically definite or probable vasculitic neuropathy), according to established guidelines [1]. An experienced neuropathologist analyzed all biopsy specimens. Exclusion criteria were the identification of a systemic vasculitis by clinical or laboratory measures or incomplete follow-up data of less than six months (Fig. 1). To rule out systemic vasculitis, patients underwent clinical investigations focusing on signs, stigmata, and predisposing factors for other-organ involvement, and laboratory testing, including differential blood count, CRP, Erythrocyte Sedimentation Rate (ESR), and cerebrospinal fluid analysis.

### Data analysis

The patients’ medical data were investigated. The clinical severity of each patient with NSVN was evaluated clinically and electrophysiologically (Tables 1 and 2). Based on the initial therapy regimen, patients were divided into four groups: (1) oral corticosteroid monotherapy, (2) pulsed intravenous corticosteroid therapy, (3) cyclophosphamide monotherapy, or (4) combination therapy, i.e., a regimen that combined different immunosuppressants. At first, data of patients who stopped their treatment due to adverse events were not included in the analysis of treatment efficacy, i.e., relapse-free time, to minimize subjective influencing factors. Besides this analysis, these patients’ data were included for further analysis. Mean age, clinical severity, and disease duration before therapy started were compared between induction therapy groups. Further, an analysis of clinical progression for 12 months was performed for each group: The relapse-free survival probability was measured from the start of the induction therapy to the time of the first relapse. According to established guidelines, relapse was defined as a progression in sensory loss or weakness, worsening disability scores, or electrophysiological parameters. Pain alone was not classified as a relapse. As additional outcome variables, changes of motor symptoms evaluated by the Medical Research Council (MRC) sum score, sensory symptoms, pain, and electrophysiological measures (N. tibialis amplitude) from baseline to follow-up (maximum of 12 months or at the point of a relapse) were included. Changes were listed as improved, unchanged, or worse. MRC sum scores and tibialis amplitudes were obtained as the mean and interquartile range at baseline and follow-up.

**Table 1:** Demographic and clinical data of patients

	CS MONO	CYC MONO	CS PULSE MONO	Combination therapy	*P*-value
Count (n)	13	10	9	5	
Sex (n) (male/female)	11/ 2	7/3	5/4	3/2	
Age (years)	64 (52.5, 73)	70 (58.75, 77.25)	68 (60, 75.5)	59 (44.5, 74)	0.318
Pallesthesia (0–8)	2 (0, 3.5)	2 (0, 3.25)	2 (0, 3.5)	2 (0, 3.5)	0.998
CMAP tibial [mV]	0.4 (0.05, 1.05)	0.4 (0.075, 1)	1.3 (0.45, 3.8)	1.8 (0.3, 14.9)	0.148
SNAP sural [µV]	0.1 (0, 3.5)	0 (0, 2.25)	2 (0, 4.5)	0 (0, 0)	0.208
Disease duration before therapy (years)	1.5 (1, 4)	2 (1, 3.5)	2 (2, 3)	1 (0.79, 3)	0.299

Values are expressed as the median and interquartile range (Q1, Q3). CS MONO: oral corticosteroid monotherapy; CYC MONO: cyclophosphamide monotherapy; CS PULSE MONO: pulsed intravenous corticosteroid monotherapy; combination therapy: therapy regimens combining immunosuppressants. Kruskal–Wallis tests were used to compare the data of each group

Results with *p* < 0.05 were considered significant

**Table 2:** Characteristics and outcome variables in treated patients with NSVN

	**CS Mono**	**CYC Mono**	**CS Pulse Mono**	**Combination**
*MRC sum score*				
a) Median (Q1, Q3) baseline/ follow-up	29 (28, 30)	28 (26, 29)	30 (28.5, 30)	28 (24.5, 30)/28 (27, 30)
b) Change at follow-up				
Improved (n)	0/13	3/10	0/9	1/5
Unchanged (n)	11/13	4/10	9/9	4/5
Worse (n)	2/13	3/10	0/9	0/5
*Sensory symptoms*				
(Change at follow-up)				
Improved (n)	2/13	2/10	2/9	3/5
Unchanged (n)	8/13	7/10	5/9	2/5
Worse (n)	3/13	1/10	2/9	0/5
*Pain*				
(Change at follow-up)				
Improved (n)	2/13	0/10	3/9	0/5
Unchanged (n)	7/13	10/10	5/9	5/5
Worse (n)	4/13	0/10	1/9	0/5
*Tibial CMAP-amplitude*				
Median (Q1, Q3) baseline/ follow-up	0.4 (0.05, 1.05)/0.5 (0.0, 0.9)	0.4 (0.075, 1)/0.25 (0.2, 0.6)	1.3 (0.45, 3.8)/0.6 (0.4, 1.4)	1.8 (0.3, 14.9)/3.7 (0.15, 14.25)
*EMG-spontaneous activity at baseline*				
Not existent (n)	5/13	2/10	5/9	1/5
Mild (n)	5/13	4/10	2/9	2/5
Moderate to severe (n)	3/13	4/10	2/9	2/5

Values are expressed as the median and interquartile range (Q1, Q3) or counts, with (n) representing the number of patients. CS Mono: oral corticosteroid monotherapy; CYC Mono: cyclophosphamide monotherapy; CS Pulse Mono: pulsed intravenous corticosteroid monotherapy; Combination: therapy regimens combining immunosuppressants. MRC (Medical Research Council) sum score was used to rate the muscle strength; follow-up medians of MRC sum scores were only listed if changed

### Statistical analysis

Statistical analysis was performed using SPSS Statistics 27 (IBM, Armonk NY, US). The Kruskal–Wallis test was used to compare the clinical and demographic data of each group. Kaplan–Meier curves were created and compared by the log-rank test to determine the relapse-free survival probability for each group. Results are expressed as medians and interquartile range. Predictors of relapse were evaluated using logistic regression; the results were expressed as odds ratios (OR) with 95% confidence intervals (95% CI) and *p*-values. Results with *p* < 0.05 were considered significant.

## Results

212 patients with the coding ICD-10 G62.88 as their primary diagnosis were identified. Of which, 62 patients were diagnosed with a pathologically confirmed vasculitic neuropathy. 43 of all vasculitic neuropathies were assigned as NSVN. 19 patients were excluded due to missing follow-up data or being diagnosed with systemic vasculitis. Initially, six patients with NSVN were also excluded due to treatment-associated side effects leading to treatment discontinuation. Thus, 37 patients were assigned to different induction treatment groups (Fig. 1): oral corticosteroid monotherapy, pulsed intravenous corticosteroid therapy, cyclophosphamide monotherapy, or combination therapy. Cyclophosphamide was administered as a pulsed therapy every 4–6 weeks (600 mg/m^2^) over 12 months unless a relapse occurred.

The clinical and demographic characteristics of all NSVN patients are provided in Table 1. There was no significant difference between the treatment groups concerning age, clinical investigations, electrophysiological measures, and disease duration before treatment initiation, proving eligibility for further comparison. All patients with NSVN showed severe axonal impairment of motor and sensory nerves in electrophysiological studies (CMAP tibial [mV]: 0.5 (0.1, 2.15), SNAP sural [µV]: < 0.0001 (< 0.0001, 2.5)). Most patients (64.9%) exhibited pathological spontaneous activity on electromyography as a sign of florid axonal damage (Table 2). At the first presentation, eight patients with NSVN depended on a walking aid. The remaining patients were able to walk independently.

**Fig. 2:**
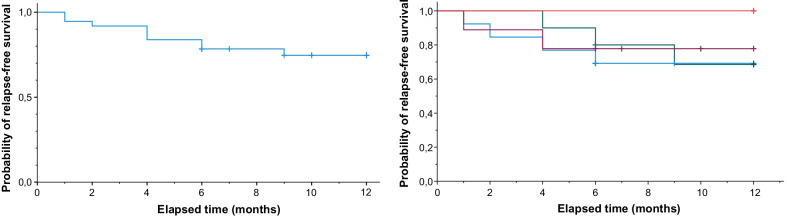
Probability of relapse-free survival in patients with non-systemic vasculitic neuropathy (NSVN). The left side shows the Kaplan–Meier curve of all treated patients combined, and the right side shows Kaplan–Meier curves for different treatment groups separately (red: combination therapy; green: cyclophosphamide monotherapy; purple: pulsed intravenous corticosteroids; blue: oral corticosteroid monotherapy). Relapse-free survival was compared by Kaplan–Meier curves and log-rank test. No significant differences were detected between treatment groups (*p* = 0.58). 24.32% of all patients relapsed within 12 months

24.3% of all patients with NSVN relapsed within 12 months (Fig. 2). The median follow-up time was 10 months (6, 12). The comparison of the different treatment groups did not detect any significant differences in relapse-free survival (*p* = 0.58; oral corticosteroid monotherapy: 4/13, CYC monotherapy: 3/10, pulsed intravenous corticosteroid therapy: 2/9, combination therapy: 0/5). No relapses were detected in the combination therapy group, whereas in all other treatment groups, relapses occurred. However, this finding was not statistically significant (*p* = 0.58). Relapses usually occurred early after treatment initiation (time to relapse [months]: 4 (1.5, 6)) with no significant differences between the treatment groups (*p* = 0.21). Further outcome measures are provided in Table 2. Four patients showed an improvement, and five patients showed a deterioration of motor symptoms evaluated by the MRC sum score during treatment. Motor symptoms of 28 patients (75.7%) were unchanged at follow-up. Similarly, nine patients showed an improvement, and six patients showed a deterioration of sensory symptoms. In 22 patients, sensory symptoms were unchanged. Pain improved in five patients treated with corticosteroids (either oral or pulsed therapy) and worsened in five patients. Pain was unchanged in all patients treated with cyclophosphamide, monotherapy or combination therapy. Electrophysiological measures only showed non-significant changes from baseline to follow-up. No patient in the combination therapy group deteriorated in one of the listed outcome measures.

In all groups, treatment-related side effects were rare but led to discontinuation of the current treatment (oral corticosteroid monotherapy (1): psychosis; CYC monotherapy (1): vomiting; pulsed intravenous corticosteroid therapy (3): tachycardia, nausea, drug intolerance; combination therapy (corticosteroids + azathioprine) (1): abdominal discomfort, restlessness). The inclusion of patients with treatment-associated side effects did not influence the significance level of relapse-free survival between the treatment groups.

As a second-line treatment, variable regimens were identified, with cyclophosphamide monotherapy being the most common (4/9 patients). Combination therapy, which included cyclophosphamide, was identified in three additional patients. Others also included intravenous immunoglobulins and methotrexate.

Biopsy specimens showed inflammatory cells in vessel walls and axonal nerve damage of a multiplex type. Necrosis or thrombosis of vessels were not observed. Analysis of nerve biopsies of all patients with NSVN showed T-cell-dominated infiltrates (21 patients with a CD4-dominated infiltrate, six patients with a CD8-dominated infiltrate, and 10 patients were classified as equal distribution of CD4 and CD8 cells). 12 specimens showed expression of the CD20 antigen. However, CD20 antigens were confined to a few lymphocytes only. Neither the immunophenotype of NSVN according to the nerve biopsy nor clinical measures predicted a relapse sufficiently (Additional file 1).

## Discussion

In this single-center retrospective study, we evaluated the efficacy of different immunosuppressive therapy regimens commonly used in clinical practice for the initial treatment of NSVN. Our data indicate that immunosuppressive therapy in NSVN is effective and mainly well tolerated. We could not detect significant differences in relapse rates between the different treatment regimens. However, there was a non-statistically significant superiority for treatment approaches combining different immunosuppressive agents, as no relapses occurred in this small subgroup. Accordingly, no deterioration of motor or sensory symptoms occurred in this group. Overall, most patients showed stabilization of clinical symptoms during treatment.

The evaluation of therapy regimens used in clinical practice in NSVN is of particular interest as no controlled therapy studies exist for this disease entity, and decisions on first-line therapy show significant variability among clinicians. Treatment recommendations are derived from the treatment of systemic vasculitis, other retrospective data analyses in NSVN, and clinical experience [2, 4, 13]. Recently, as the initial treatment, combination therapy was suggested to avoid relapses [2]. This therapy regimen included corticosteroids and cyclophosphamide or methotrexate. However, this approach yields the risk of more treatment-associated complications compared to monotherapy, at least considering the need for long-term immunosuppressive treatment. Moreover, there is an increased concern of susceptibility to infectious diseases, especially in the light of a pandemic [10].

Other retrospective studies also showed the efficacy of immunosuppressive treatment in NSVN [4, 13]. However, the necessary initial treatment escalation remained unclear. Collins et al. reported reduced relapse rates with treatments combining cyclophosphamide and corticosteroids; nevertheless, in a different cohort of 60 patients with NSVN, no relapse occurred in patients treated with corticosteroid monotherapy [4, 13].

Monotherapy might be sufficient for most patients, considering our data. Moreover, our findings do not suggest an overall benefit of treatment initiation with cyclophosphamide over corticosteroids when started as a monotherapy. This observation aligns with the 2010 Peripheral Nerve Society NSVN guidelines that recommended either corticosteroid monotherapy or combination therapy with cyclophosphamide in severe NSVN cases [1].

Rituximab has been discussed as an alternative to cyclophosphamide for severe NSVN cases. This proposition is mainly based on evidence from ANCA-associated systemic vasculitis, where rituximab was not inferior to cyclophosphamide to induce remission [11]. However, it is not known if these results can be extrapolated to NSVN. Moreover, our data show, similar to other studies, B-cells are uncommon in nerve biopsies of patients with NSVN, and inflammatory infiltrates are mainly composed of CD4 and CD8 positive T-cells [3, 7, 12]. These findings suggest the use of cyclophosphamide rather than rituximab.

In our cohort, 24.3% of all patients relapsed despite immunosuppressive therapy, emphasizing the importance of appropriate treatment. Combining data analysis of several other studies showed similar relapse rates in treated patients [2]. As relapses usually occurred early after treatment initiation in our cohort, close follow-up investigations are needed, especially as we could not identify significant predictors for relapse. Similarly, in a cohort of 48 patients, no clinical or laboratory parameters predicted corticosteroid responsiveness [4]. In ANCA-associated vasculitis, lung involvement was associated with an increased risk of relapse. However, nerve biopsy findings were not investigated [5, 9].

As a second-line therapy, cyclophosphamide monotherapy was preferentially used. However, our cohort’s second-line treatment approaches were inconsistent, as seen in other case series where various immunosuppressive agents were applied in refractory vasculitic neuropathy cases [8].

### Limitations

Despite the relevant number of screened patients, the numbers within the different treatment groups were low. Therefore, treatment effects might not have been adequately represented, especially concerning treatment approaches combining different immunosuppressants. Moreover, a prospective study design would be preferable to evaluate effects on relapse-free survival, including recent developments in vasculitis therapy, i.e., rituximab or other monoclonal antibodies. However, as these studies do not exist now, combining data from different clinical cohorts may help improve the care of patients with NSVN and avoid unnecessary or inappropriate treatment.

Our study was not designed to evaluate the efficacy of second-line therapy. More data on maintenance therapy and the long-term effects of immunosuppressive treatment in NSVN are needed.

## Conclusion

Our data show that induction therapy in NSVN used in clinical practice is effective and mainly well tolerated. Overall, our investigations suggest that a treatment approach with oral corticosteroids should be considered first-line therapy. In severe cases or when relapse occurs, combination therapy, including CYC, should be considered. Close follow-up is mandatory to identify patients who relapse.

## Supplementary Information

**Additional file 1:** Predictors of relapse in patients with NSVN.

## Data Availability

The data supporting this study’s findings are stored on a secured server at the University Hospital of Cologne, Germany, and are available from the corresponding author upon reasonable request.
