# Supplementary material for: Single-center experience of induction therapy in non-systemic vasculitic neuropathy
Source: Neurol Res Pract. 2022 Aug 15;4:32. doi: 10.1186/s42466-022-00198-5 (PMC9377119; doi:10.1186/s42466-022-00198-5)
Supplement: Supplementary file 1 — Additional file 1: Predictors of relapse in patients with NSVN. [file 42466_2022_198_MOESM1_ESM.pdf]

Biopsy results and OR

Predictor	P	OR (95% CI)
CD4-dominated biopsy	0.933	0.938 (0.207 – 4.256)
CD8-dominated biopsy	0.577	1.714 (0.258 – 11.401)
CD4/CD8-equal biopsy	0.710	0.714 (0.121 – 4.204)
CD20-positive cells in biopsy	0.947	1.056 (0.214 – 5.211)
Disease duration before therapy	0.177	0.658 (0.358 – 1.209)
CMAP (tib.) of more affected lower limb [mV]	0.273	0.873 (0.684 – 1.113)
SNAP (sur.) of more affected lower limb [µV]	0.501	1.106 (0.824 – 1.484)
